# Translation and psychometric properties of the Chinese (Mandarin) version of the Child Oral Health Impact Profile-Short Form 19 (COHIP-SF 19) for school-age children

**DOI:** 10.1186/s12955-014-0169-1

**Published:** 2014-11-30

**Authors:** Chenghao Li, Bin Xia, Yu Wang, Xuelin Guan, Junwei Yuan, Lihong Ge

**Affiliations:** Department of Pediatric Dentistry, Peking University School and Hospital of Stomatology, Zhongguancun South Avenue 22, Haidian District, Beijing, 100081 China; Center of Stomatology, Peking University Hospital, Yiheyuan Road 5, Haidian District, Beijing, 100871 China

**Keywords:** Quality of life, Oral health, Reliability, Validity, School-age, Children

## Abstract

**Background:**

Although caries and malocclusion occur with a high prevalence in Chinese school-age children, there were no appropriate instrument to assess the oral health-related quality of life (OHRQoL) for this population. The aim of our study was to develop a Chinese (Mandarin) version of the Child Oral Health Impact Profile-Short Form 19 (COHIP-SF 19) and provide a preliminary test of its psychometric properties.

**Methods:**

The Chinese version of COHIP-SF 19 was developed through a standard translation and back translation procedure. The psychometric properties of the instrument were tested among 644 school-age children in Beijing, China, including the internal consistency, test-retest reliability, discriminant and convergent validity. A Mann-Whitney U test was used to determine the capability of the instrument to differentiate children with different caries and malocclusion outcomes. And partial Spearman correlations were used to determine the relationships between the OHRQoL scores and clinical-severity indicators and self-perceived health ratings, respectively.

**Results:**

Chinese school-age children had relatively high OHRQoL scores, in spite of the fact that oral impacts were quite common (56.3%). The internal consistency and retest reliability were good to excellent with a Chronbach’s alpha of 0.81 and an intra-class correlation coefficient (ICC) of 0.77. Children who had active tooth decay or severe malocclusion had significantly lower COHIP-SF 19 scores (*P* ≤0.001). Girls had somewhat higher scores in the oral health and functional well-being subscales (*P* <0.05), while children from rural districts had lower scores than children from urban areas (*P* <0.05). We observed a low to moderate correlation between the overall COHIP-SF 19, subscale scores and clinical severity indicators as well as self-perceived health ratings, after adjustment for children’s age, gender, and school district (│*r*_*s*_│ =0.11 - 0.51, *P* <0.05).

**Conclusion:**

We confirmed satisfactory psychometric properties for the Chinese version of COHIP-SF 19 in a community sample of Chinese school-age children. The OHRQoL instrument should play a more important role in future clinical studies, epidemiological surveys and potential public health policy in China.

## Background

The oral health-related quality of life (OHRQoL) is a multidimensional construct which contains a subjective assessment of an individual’s oral condition, functional and emotional well-being, expectations and satisfaction with care, and sense of self [1]. Over the past 30 years, it has become important for the evaluation of a person’s oral health outcomes through subjective estimation [2]. OHRQoL evaluation can be supplementary to traditional medical/dental criteria for needs assessment and health care outcomes for a population or a specific clinical group [3]. OHRQoL instruments can be used in clinical practice and research to focus on a person’s social and emotional experience and physical function [4]. OHRQoL instruments can also be used in epidemiological studies to perform thorough needs assessments for both general and vulnerable populations. Such assessments can provide essential information regarding public health issues relevant to specific populations like school-age children [5]. Therefore, incorporating OHRQoL instruments into traditional clinical indicators can produce important benefits for individual patients, community-based dental practices, clinical research, and potential public health policy [1].

Various instruments for the measurement of OHRQoL have been developed since 1990s [6]. However, the development of instruments for children’s OHRQoL has been slower compared with those for adults. The first comprehensive measure of children’s OHRQoL did not exist until 2002 [7]. Owing to their immature and developing skills and functions (e.g., abstract reasoning skills and cognitive functions), it can often be difficult to understand and assess children [8]. Due to their rapid physical and mental growth and cognitive changes, several age-specific instruments have been developed for children, including the Child Perception Questionnaire (CPQ) [7], the Early Childhood Oral Health Impact Score (ECOHIS) [9], Pediatric Oral Health-related Quality of Life (POQL) [10],Child Oral Impacts on Daily Performance (Child-OIDP) [11], the Scale of Oral Health Outcomes for 5-year-old children (SOHO-5) [12], and the Child Oral Health Impact Profile (COHIP) [13].

Although great effort has been devoted to measurement of children’s OHRQoL during the past decade all over the world [4,9,14], only a few studies have evaluated subjective indicators in children and their validation in China [15-17]. Meanwhile, the limited studies have primarily targeted preschool children (3-5 years) with ECOHIS [16] and adolescents (11-14 years) with CPQ [17]. Investigations focusing on the OHRQoL of school-age children are lacking in China. Combined with the fact that certain groups of children have suffered from severe oral disease, including caries and malocclusion [18,19], there is a significant need to characterize the OHRQoL in this population.

The COHIP was developed to assess children’s oral-facial well-being with a wide range of age (8-15 years) and across ethnicities, health systems, and various conditions [20,21]. It contains 34 questions and 5 subscales (oral health, functional well-being, socio-emotional well-being, school environment, and self-image). One important characteristic of the scale is the inclusion of positive aspects of OHRQoL (e.g., confidence and attractiveness). The reliability and validity of the COHIP were tested and phychometric properties were confirmed [20,22]. In addition to the original versions in English, Spanish, and French, the COHIP has been translated into Dutch [23], Korean [24] and Persian [25], and was shown to be reliable and valid in cross-cultural adaptation. To adapt this system for clinical research and epidemiological studies, a shortened instrument, the Child Oral Health Impact Profile-Short Form 19 (COHIP-SF 19), was developed in 2012 [26]. The COHIP-SF 19 was shortened to 19 items and 3 subscales (oral health, functional well-being, and socio-emotional well-being) to maintain appropriate psychometric properties. Additionally, the lower limit of participant age was reduced to 7 years.

Because of linguistic difference and cross-cultural issues, OHRQoL instruments must not only be translated but also validated in the target population prior to cross-cultural and cross-national adaptation [27-30].

Our study aimed to develop an appropriate Chinese (Mandarin) version of COHIP-SF 19 and to assess the reliability and convergent and discriminant validity of the Chinese version of COHIP-SF 19 in school-age children. Furthermore, a description of the OHRQoL for the target population is presented here as well.

## Methods

### Translation of COHIP-SF 19

The original English version of COHIP-SF 19 was obtained by the developer Dr. Broder [13] and was translated and adapted according to standard guidelines [31]. The scale was first translated into Chinese (Mandarin) by a pediatric dentistry post-graduate and a pediatric dentist who were both fluent in Chinese and English. The translation was assessed and revised by an expert panel with regard to concept and item equivalence between the original version and Chinese version. The panel consisted of researchers, two pediatric dentistry experts, one Public Health expert familiar with quality of life questionnaires and a Chinese scholar majoring in the English language. Attention was given to the concept of the words in the different languages in order to produce a similar impression on respondents in both cultures and identify possible difficulties in understanding the questionnaires. The consensus translation was then pilot-tested on a sample of 38 children of appropriate age and their caregivers to determine its sensitivity to Chinese culture and the selection of proper wording. With minor modifications, the consensus version was translated back into English by a Chinese scholar majoring in English language and literature and a lay bilingual person who had lived in the US for 15 years. Both of the translators were not familiar with the questionnaire. The backward-translated English version was then re-evaluated by Dr. Broder and the expert panel and verified to be nearly the same as the original English version. Finally, after minor modifications, the Chinese version of COHIP-SF 19 was confirmed by the expert panel.

### Measurement of variables

The Chinese version of the COHIP-SF 19 questionnaire consisted of 19 questions forming 3 conceptual subscales: oral health (5 items), functional well-being (4 items), and socio-emotional well-being (10 items). Two of the items were positively worded questions. Children were asked how often they had experienced oral impacts during the past 3 months and each question was answered with a five-point Likert scale ranging from ‘never’ to ‘almost all the time’. Responses to the two positively worded questions were recorded as ‘never’ =0, ‘almost never’ =1, ‘sometimes’ =2, ‘fairly often’ =3, and ‘almost all the time’ =4. Scoring for the 17 negatively worded items was reversed. As a result, higher COHIP-SF 19 scores reflected a more positive OHRQoL [20]. The overall COHIP-SF 19 score was calculated by summing all 19 items scores within a range of 0-76. Additionally, there were two self-rated items concerning health/oral health that were scored from ‘excellent’ to ‘very bad’ and one item addressing the preceived need for dental treatment that was rated from ‘strongly disagree’ to ‘strongly agree’.

### Psychometric testing of the scale

This cross-sectional study was conducted in Beijing, the People’s Republic of China, with a target population of children from 7- to 13-year-old enrolled in public primary schools. The study was approved by the ethics committee of the Peking University School and Hospital of Stomatology (No. PKUSSIRB-201412014). Nonrandom samples were used to collect data. As was suggested by *Charter* [32], the minimum sample size needs to be larger than 400 to evaluate reliability and validity. In accordance with previous studies [20,25], a sample size of 600 was chosen. Two urban schools and two rural schools were invited to participate in the study. Both the two urban schools and one of the rural schools accepted the invitation. For the time limitation, the two urban schools agreed to select one class from each grade (grade 2-6) to be investigated. While the rural school agreed to select two classes from each grade (grade 2-6). Children were excluded if they had low literacy skills, mental disability, or underwent orthodontic treatment. Therefore, 644 sutdents were initially enrolled in the study.

After describing the purpose of the study, the consent of each child and guardian was obtained prior to the study. The consent of the children had precedence over parental consent. A self-administrated questionnaire was completed by each child. Two trained research assistants were available to provide assistance for younger children, if needed. Pictures and a cue sheet were also available for younger children to help them comprehend the questions. All participants completed the questionnaire within 6-7 minutes. The same questionnaire was used 2-3 weeks later on 159 (aprroximately 25%) of the participants for the purpose of estimating retest reliability.

Following completion of COHIP-SF 19, each child received a dental examination performed by one of the four calibrated dentists. The four dentists achieved satisfactory agreement prior to the project. Kappa values for the examination of caries were from 0.60 to 0.75 inter-examiner and from 0.66 to 0.88 intra-examiner. According to the intra-class correlation coefficient (ICC), inter- and intra-examiner reliability was between 0.84 to 0.94 and 0.77 to 0.92, respectively, for malocclusion examination. As the target population had different types of dentition, the number of carious deciduous (dt) and permanent (DT) teeth was recorded according to World Health Organization (WHO) criteria [33]. The number of carious teeth was subsquently dichotomized into ‘with active decay’ (DT + dt >0) and ‘without active decay’ (DT + dt =0). For malocclusion examination, occlusal traits were examined. In concordance with previous epidemiological surveys [18,34,35], children with no malocclusion traits were categorized as ‘no malocclusion’. Children were diagnosed with ‘severe malocclusion’ if they possessed one or more of the following traits: Angle Class III, overbite >3 mm, overjet >5 mm, impinging bite, open bite, anterior crossbite, posterior crossbite, scissor bite, ectopic eruption of first molar, or crowding >4 mm. The remaining children were categorized as ‘mild malocclusion’. For the purpose of data analysis, the groups ‘no malocclusion’ and ‘mild malocclusion’ were considered to be ‘without orthodontic treatment need’, and the ‘severe malocclusion’ group was defined as ‘with orthodontic treatment need’. Clinical examinations were performed according to WHO criteria for visual dental investigation in classrooms [33]. Small monetary incentives were offered for participants’ effort and time.

Prior to data analysis, participants that did not complete more than 75% of responses were excluded. On the subscale level, if more than two-thirds of the items were missing in a certain subscale, the sample was catagorized as missing. If fewer items were missing, the missing values were replaced using the mean of available items.

The COHIP-SF 19 impact was defined as rating 17 negatively worded items as ‘fairly often’ or ‘almost all the time’ or rating 2 positively worded items as ‘almost never’ or ‘never’.

The distribution of overall COHIP-SF 19 and each subscale scores was tested using the Kolmogorov-Smirnov test.

Internal consistency for COHIP-SF 19 was measured using Chronbach’s alpha, corrected item-total correlation and Chronbach’s alpha if an item was deleted. Test-retest reliability was indexed by the ICC.

Discriminant validity of the scale was assessed by comparing the mean total scores across the ‘with/without active decay’ and ‘with/without orthodontic treatment need’ groups. Discriminant validity was further evaluated by examing the association between the COHIP-SF 19 scores and clinical severity with the number of decayed teeth (DT + dt) and the categories of malocclusion (no/mild/severe malocclusion) adjusted by age, gender, and school district.

Convergent validity was assessed by examining the relationship between COHIP-SF 19 scores and the rating of self-perceived health/oral health and dental-treatment need after controlling for demographic covariates.

The non-parametric Mann-Whitney U test and partial Spearman correlation were used. SPSS version 20 was used for analysis. The significance level was set at *p* <0.05.

## Results

### Descriptive statistics

In all, 644 children were selected to participate the study. Ten parents and one child declined to provide consent for a response rate 98.3%. Eight participants undergoing orthodontic treatment were excluded from the analysis procedure. Also, 5 students who were outside of the age range (less than 7 years old) were not included. Finally, of 620 participants, 53.2% (n =330) were boys between the ages of 7 to 13 with a mean age of 9.1 ± 1.5 years. Three hundred and ninety-one students (63.1%) were from rural districts. None of the cases were dropped due to incomplete data.

The mean COHIP-SF 19 score was 62.2 ± 8.2 and the median was 64 (range 25-76). Mean, median, range, and quartiles for all COHIP-SF 19 responses and each subscale score are shown in Table 1. Of all participants, 56.3% experienced one or more COHIP-SF 19 impacts, with one or more items rated ‘fairly often’ or ‘almost all the time’ for the 17 negatively worded items and ‘almost never’ or ‘never’ for the 2 positively worded items. The impact prevalences for subscales were 35.8% (socio-emotional well-being), 34.2% (oral health), and 12.7% (functional well-being), respectively.

**Table 1:** **Descriptive statistics for COHIP-SF 19 and subscale scores (n =620)**

**Scale (possible range)**	**Mean (SD)**	**Median (range)**	**Quartiles**	
			**25th**	**75th**
Overall COHIP-SF 19 (0-76)	62.2 (8.2)	64 (25-76)	57	68
Oral health (0-20)	14.4 (3.3)	15 (3-20)	12	17
Function well-being (0-16)	13.7 (2.3)	14 (3-16)	13	16
Socio-emotional well-being (0-40)	34.2 (4.5)	35 (12-40)	32	38

Of all participants, 72.4% (n =449) and 44.0% (n =273) reported that their general health or oral health was excellent or good, respectively, while 43.5% (n =270) perceived that they needed dental treatment and 31.3% (n =194) reported no need for dental treatment.

The Kolmogorov-Smirnov test showed that the distribution of overall COHIP-SF 19 scores was positively skewed and significantly different from a normal distribution (skewness = -0.96; kurtosis =1.34; *P* <0.001) as were the subscales.

### Reliability

The internal consistency for the overall COHIP-SF 19 score was excellent with a Chronbach’s alpha of 0.81, good for the socio-emotional well-being subscale (0.74), and moderate for the other two subscales (0.56-0.59; Table 2). The corrected item-total correlations were all positive and ranged from 0.28 to 0.53 for COHIP-SF 19 and the subscales. The Chronbach’s alpha value did not increase if any of the items were deleted. In terms of test-retest reliability, the ICC was 0.77 for the overall COHIP-SF 19, revealing good retest reliability. Meanwhile, the retest reliability was excellent for the oral health subscale, and good for the functional and the socio-emotional well-being subscales, with ICC values of 0.81, 0.74 and 0.62, respectively.

**Table 2:** **Internal reliability analysis of COHIP-SF 19 and each subscale (n =620)**

**Scale (number of items)**	**Chronbach’s alpha**	**Corrected item-total correlation**	**Alpha if an item is deleted**
Overall COHIP-SF 19 (19)	0.81	0.29-0.53	0.79-0.81
Oral health (5)	0.59	0.28-0.41	0.49-0.56
Functional well-being (4)	0.56	0.33-0.37	0.47-0.50
Socio-emotional well-being (10)	0.74	0.29-0.53	0.70-0.74

### Discriminant validity

The comparison of COHIP-SF 19 and each subscale score with different clinical outcomes (active decay or a normative need for orthodontic treatment) is presented in Table 3. Children with no active decay (DT + dt =0) had significantly higher overall score for COHIP-SF 19 (*P* <0.001) and all three subscales (*P* ≤0.002) by the Mann-Whitney U test. Children without a need for orthodontic treatment showed significantly higher overall scores for COHIP-SF 19 and the two subscales (*P* ≤0.004), with the exception of the functional well-being subscale (*P* =0.166).

**Table 3:** **Comparison of COHIP-SF 19 and each subscale scores by the different clinical outcomes of caries and malocclusion (n =620)**

	**Overall COHIP-SF 19**	**Oral health**	**Functional well-being**	**Socio-emotional well-being**
	**Mean (SD)**	**Mean (SD)**	**Mean (SD)**	**Mean (SD)**
No active decay (n = 248)	64.4 (7.2)	15.4 (2.7)	14.1 (2.1)	34.9 (4.1)
Active decay (n = 372)	60.8 (8.5)	13.7 (3.5)	13.4 (2.5)	33.7 (4.7)
*P*-value	<0.001	<0.001	=0.002	=0.001
Without orthodontic treatment need (n =176)	64.0 (7.7)	15.0 (3.1)	13.8 (2.5)	35.2 (4.1)
With orthodontic treatment need (n =444)	61.5 (8.3)	14.1 (3.3)	13.6 (2.3)	33.8 (4.6)
*P*-value	=0.001	=0.004	0.166	<0.001

Mann-Whitney non-parametric statistics were used.

Discriminant validity was further addressed by examing the relationships between clinical severity indicators and overall COHIP-SF 19 and subscale scores, after controlling for participant age, gender, and school district (Table 4). The number of actively decaying teeth (DT + dt, range =0 - 16) was significantly negatively correlated with the overall COHIP-SF 19 and all three subscale scores (*P* ≤0.002), although the relationships were weak (│*r*_*s*_│ =0.12 - 0.21). Weak but statistically significant relationships were also found between the categories of malocclusion and the overall COHIP-SF 19 and two subscale scores (│*r*_*s*_│ =0.11 - 0.17, *P* ≤0.009), with the exception of the functional well-being subscale (*P* =0.339).

**Table 4:** **Partial Spearman correlations** ^*^ **between clinical severity indicators and the overall COHIP-SF 19 and subscale scores (n =620)**

	**Caries indices (DT + dt)**		**Categories of malocclusion (no/mild/severe malocclusion)**	
	***r***_***s***_	***P*** **-value**	***r***_***s***_	***P*** **-value**
Overall COHIP-SF 19	−0.21	<0.001	−0.13	=0.001
Oral health	−0.21	<0.001	−0.11	=0.009
Functional well-being	−0.12	=0.002	−0.04	=0.339
Socio-emotional well-being	−0.14	=0.001	−0.17	<0.001

^*^The partial spearman correlations were adjusted by age, gender and school district.

Table 5 displays the comparison of the COHIP-SF 19 scores with demographic variables. No statistical difference was found between the two different age groups (7-9 and 10-13 years). Girls had higher COHIP-SF 19 and subscale scores than boys, but significant differences were only observed in the oral health and the functional well-being subscales (*P* =0.026 and 0.004, respectively). Children from rural districts had significantly lower scores in the overall COHIP-SF 19 (*P* =0.005), the oral health subscale (*P* <0.001), and the functional well-being subscale (*P* =0.039) than participants from urban districts, but this was not true for the socio-emotional well-being subscale.

**Table 5:** **Descriptive analysis of COHIP-SF 19 and each subscale score regarding age, gender, and school district**

	**Age**			**Gender**			**School district**		
	**Mean (SD)**		***P*** **-value**	**Mean (SD)**		***P*** **-value**	**Mean (SD)**		***P*** **-value**
	**7-9 y**	**10-13 y**		**Male**	**Female**		**Urban**	**Rural**	
Overall COHIP-SF 19	61.7 (8.5)	62.9 (7.8)	=0.085	61.6 (8.7)	63.0 (7.5)	=0.075	63.6 (7.3)	61.5 (8.6)	=0.005^§^
Oral health	14.1 (3.5)	14.7 (3.0)	=0.093	14.0 (3.5)	14.7 (3.0)	=0.026^*^	15.1 (2.7)	13.9 (3.5	<0.001^§^
Functional well-being	13.5 (2.6)	14.0 (2.0)	=0.126	13.4 (2.5)	14.0 (2.1)	=0.004^§^	14.0 (1.9)	13.5 (2.5)	=0.039^*^
Socio-emotional well-being	34.1 (4.5)	34.3 (4.5)	=0.506	34.1 (4.7)	34.3 (4.3)	=0.974	34.4 (4.5)	34.0 (4.5)	=0.208

Mann-Whitney non-parametric statistics were used.

^*^significant at *P* <0.05, ^§^significant at *P* <0.01.

### Convergent validity

All of the partial correlations were significant, and all of the coefficients were positive (range =0.10 - 0.51). The highest partial correlation coefficients were between the overall COHIP-SF 19 and the oral health subscale scores and perceived oral health ratings, with values of 0.48 and 0.51, respectively. The lowest partial correlation coefficient was between the socio-emotional well-being subscale score and perceived dental treatment need (*r*_*s*_ =0.10). The coefficients of the partial Spearman correlations are shown in Table 6.

**Table 6:** **Partial Spearman correlations** ^*^ **of self-perceived assessment** ^**§**^ **with the overall COHIP-SF 19 and each subscale scores (n =620)**

	**Perceived general health**		**Perceived oral health**		**Perceived treatment need**	
	***r***_***s***_	***P*** **-value**	***r***_***s***_	***P*** **-value**	***r***_***s***_	***P*** **-value**
Overall COHIP-SF 19	0.36	<0.001	0.48	<0.001	0.22	<0.001
Oral health	0.34	<0.001	0.51	<0.001	0.26	<0.001
Functional well-being	0.32	<0.001	0.36	<0.001	0.18	<0.001
Socio-emotional well-being	0.22	<0.001	0.28	<0.001	0.10	=0.011

^*^The partial spearman correlation was adjusted by age, gender, and school district.

^§^Higher scores indicate better health or lower perceived need for treatment.

## Discussion

To efficiently assess the OHRQoL for school-age children in China, a standard and validated instrument is essential [15]. The full version of COHIP has been previously validated and applied across different culture backgrounds [21,24,25]. In this study, the Chinese (Mandarin) version of COHIP-SF 19 was developed in accordance with suggested guidelines [31]. The procedure included translation, back translation, conceptual equivalence confirmation by the original developer, an expert panel and a convenience sample of children and caregivers. The Chinese version of COHIP-SF 19 was shown to have satisfactory psychometric properties for school-age children in China based on the findings of the study. The instrument was reliable and valid for the estimation of OHRQoL among Chinese schoolchildren 7-13 years in age.

COHIP-SF 19 scores from the community sample were relatively high, indicating generally good OHRQoL. The positive distribution of the scores was consistent with the findings from previous studies [20,24-26]. Nevertheless, the prevalence of COHIP-SF 19 impact was quite high (56.3%). Although different results have been reported (from 66 to 96.2%), a high prevalence of COHIP impact has been demonstrated across Asian countries [24,25]. With high incidence of caries and malocclusion in Chinese school-age children [18,19], significant impacts for children in ‘oral health’ and ‘socio-emotional well-being’ fields were not unexpected.

Psychometric tests of the Chinese version of COHIP-SF 19 were satisfactory and supplied strong support for its reliability and validity. Chronbach’s alpha for the overall COHIP-SF 19 was 0.81, close to the original study in the US (0.82-0.88) [26]. For subscales, Chronbach’s alpha values were relatively lower, especially for the oral health and functional well-being subscale (0.59 and 0.56, respectively). This could be due to the small number of items in the two subscales, with 5 items for oral health and 4 items for functional well-being [36]. The corrected item-total correlations for the 19 items were all above the minimum recommended level of 0.2 for inclusion of an item in a scale [24,29]. Meanwhile the higher alpha value did not increase when any item was deleted, relative to the original Chronbach’s alpha value. Therefore, there was no need to remove any item from the scales, and the adequate organization of the items was confirmed. The test-retest reliability for the overall COHIP-SF 19 was considered good with an ICC value of 0.77 that was very near to 0.8 and thus showed good reproducibility.

In the discriminant validity test, COHIP-SF 19 differentiated school-age children with different clinical indicators. It also showed that children with better dental health status had higher OHRQoL scores, as expected. In agreement with previous findings, children without active decay reported a higher OHRQoL than children with active decay in this study [24-26]. *Broder* et al. [26] reported that US Latino children with caries in permant teeth had significantly lower scores in the overall COHIP-SF 19 and oral health subscale. Children with or without orthodontic treatment need could also be distinguished based on the COHIP-SF 19 scores. Chinese children needing orthodontic treatment had significantly lower OHRQoL scores, in concordance with Korean and Iranian children [24,25]. In further discriminant validity exploration, a distinct gradient in the average COHIP-SF 19 score across the degrees of caries and malocclusion severity was discovered. Although the absolute values of the partial Spearman correlation coefficients were small, the findings were similar or a little higher compared to work by *Asgari* et al. [25]. The same trend was found among North American school children, but the relationships were somewhat stronger [20,26]. Alternatively, one study found that a particular OHRQoL instrument (Child-OIPD) could not distinguish the groups with or without a need for orthodontic treatment [37]. The various outcomes in different studies demonstrated that OHRQoL is a heterogeneous construct that is affected by an individual’s experiences, expectations and perceptions. A person’s responses to a valid instrument used to detect whether a physical condition affects one’s personal or social well-being are driven by complicated variables [25].

Convergent validity was proven by weak to moderate positive relationships between the COHIP-SF 19 and subscales scores and subjective ratings of health/oral health and dental treatment need, in agreement with previous studies. When OHRQoL was higher, self-reported health/oral health was also higher, and the need for dental treatment was lower [20,24,25]. Unsurprisingly, COHIP-SF 19 had a stronger relationship with the perceived oral health rating than with general health. This trait highlighted the utility of using a disease-specific instrument of quality of life to evaluate the impact of oral health conditions and concerns among children [20,37].

One of the challenges for chilren’s OHRQoL measurement is the stability of the instrument through different age groups. With the development of perception and an increase in life experience, there is potential discrepancy in OHRQoL between younger and older children [38]. Therefore, different versions of some instruments have been developed for children in different age groups [7,39]. However, some self-reported instruments have been proved to be reliable and valid when used among very young children in recent years [12,26,40]. Moreover, even 5-year-old children are believed to be capable of providing their own perceptions of oral health impacts when they use these instruments [12,40]. The COHIP-SF 19 was designed to be used with a broad age range (7-18 years) across oral conditions [26]. In the present study, no differences in OHRQoL were found between younger and older age groups, indicating that COHIP-SF 19 is also appropriate for Chinese school-age children within the broad tested age range (7-13 years). Therefore, the Chinese version of COHIP-SF 19 will be useful for future longitudinal studies, both in clinical trails and epidemiological surveys [26].

In the discriminant validity test on other demographic variables, Chinese girls had higher mean scores in the oral health and functional well-being subscales. Korean girls also showed small but significant increases in COHIP scores [24]. But the results were inconsistent in other countries [20,25]. These inconsistent results may show a distinctive characteristic for the OHRQoL assessment of children in East Asia that should be further assessed in future studies. In line with previous works [20,24], children of lower socioeconomic status from rural schools were found to have significantly lower OHRQoL scores in the overall COHIP-SF 19 and two subscales but not the socio-emotional well-being score.

A limitation in the present cross-sectional study was the unbalanced and non-random sample. Although the sample size was large enough [32], replication of the findings in a random representative sample is essential. Furthermore, longitudinal studies are required to estimate longitudinal validity and responsiveness of these measurements. Additionally, this OHRQoL instrument is intended for use in clinical trails to evaluate its sensitivity to clincial outcomes and minimal clinically important changes (e.g., malocclusion). For this purpose, a further study on the OHRQoL of Chinese school-age childern with malocclusion who are seeking treatment will be soon performed in China.

## Conclusion

The Chinese version of COHIP-SF 19 was successfully developed following a standard procedure for cross-cultural adaptation of an OHRQoL instrument. Meanwhile, the internal reliability, retest reliability, discriminant validity and convergent validity of the scale has been confirmed in a community sample of Chinese school-age children. With the high prevalence of oral impacts in school-age children and a lower OHRQoL among children from rural areas, the OHRQoL instrument should play a more important role in future clinical studies, epidemiological surveys and potential public health policy in China.

## Abbreviations

COHIP-SF 19: Child Oral Health Impact Profile-Short Form 19
OHRQoL: Oral health-related quality of life
ICC: Intra-class correlation coefficient
CPQ: Child Perception Questionnaire
ECOHIS: Early Childhood Oral Health Impact Score
POQL: Pediatric Oral Health-related Quality of Life
Child-OIDP: Child Oral Impacts on Daily Performance
SOHO-5: Scale of Oral Health Outcomes for 5-year-old children
COHIP: Child Oral Health Impact Profile
US: United States
dt: the number of decayed deciduous teeth
DT: the number of decayed permanent teeth
WHO: World Health Organization
